# Supplementary material for: Genome-wide study of linkage disequilibrium, population structure, and inbreeding in Iranian indigenous sheep breeds
Source: PLoS One. 2023 Jun 2;18(6):e0286463. doi: 10.1371/journal.pone.0286463 (PMC10237446; doi:10.1371/journal.pone.0286463)
Supplement: Attachment — Submitted filename: Responses to Reviewers Comments.docx [file pone.0286463.s001.docx]

**Manuscript Submission ID: PONE-D-22-33894 [EMID:0474543641223c89]**

**Title: " Genome-wide study of linkage disequilibrium, population structure, and inbreeding in Iranian indigenous sheep breeds", Barani et al.**

Dear Editor

We would like to thank you for giving us the opportunity to revise and improve our manuscript for possible publication in PLOS ONE. We deeply appreciate for the time and effort that the reviewers and you, taken to providing comments on our manuscript that considerably improved it.

We have provided a systemic response to each question/comment and alterations on a point by point basis as appended below. Please find Reviewer's comments in black and our responses in red color. The changes within the main text had been shown in a marked-up copy of our manuscript that highlights changes made to the original version in a separate file labeled 'Revised Manuscript with Track Changes'. Also, an unmarked version of our revised paper without tracked changes has been uploaded as a separate file labeled 'Manuscript'. We did also our best to improve the quality of writing and hopefully these changes have addressed the concerns.

Best regards,

Corresponding Author

Mohammad Hossein Moradi

**======== Reviewers' comments:
Reviewer's Responses to Questions**

1. Is the manuscript technically sound, and do the data support the conclusions?

Reviewer #1: Yes

Reviewer #2: Yes

2. Has the statistical analysis been performed appropriately and rigorously?

Reviewer #1: Yes

Reviewer #2: Yes

3. Have the authors made all data underlying the findings in their manuscript fully available?

Reviewer #1: Yes

Reviewer #2: Yes

4. Is the manuscript presented in an intelligible fashion and written in standard English?

Reviewer #1: Yes

Reviewer #2: Yes

5. **Review Comments to the Author**
**======================= Reviewer 1**

The manuscript entitled “Genome-wide study of linkage disequilibrium, population structure, and inbreeding in Iranian indigenous sheep breeds” is interesting. In the study, the authors used OvineSNP50 Bead Chip array to estimate and compare LD, genetic diversity, effective population size and genomic inbreeding of three Iranian indigenous sheep breeds. The authors found that the Baluchi breed has higher levels of LD and haplotype blocks than other two breeds. Estimation of genomic inbreeding showed the highest inbreeding coefficient in Baluchi and the lowest in Zel breed that was explained by high pressure of artificial selection on Baluchi breed. Overall, the findings are interesting and the manuscript is well structured and well written.

- The authors deeply appreciate the Reviewer for the time spending for studying our manuscript and for the positive feedback.

**Comment 1:** My only concern is that the sample size is less specially for Lori-Bakhtiari (=47) and Zel (=47) breeds. Otherwise, the manuscript has merit and may be accepted for publication.

- **Response 1:** While the authors agree to this comment of the referee that the results can be improved by increasing the sample size, the main point is that the samples used in the current study are the only genomic information available from these valuable genetic resources and we encountered financial limitations for genotyping more animals. However, as mentioned in the text, sample size correction was performed for different parameter calculations such as r^2^ and N_e_ in this study to address the referee's concern and the extend of linkage disequilibrium was obtained by r^2^ because it is less affected by sample size and MAF (compared to D`). Likewise, there are many published reports that have been performed with almost similar conditions (or even less sample size) in different indigenous sheep breeds. Examples are detailed below:
- [*BMC Genomics*](https://bmcgenomics.biomedcentral.com/), 2015, 12 up to 15 sample size from 10 Chinese indigenous sheep breeds, *https://doi.org/10.1186/s12864-015-1384-9*.
- [*Livestock Science*](https://www.sciencedirect.com/journal/livestock-science), 2020, 18 up to 34 animal samples from different Ethiopian short fat-tailed sheep breeds, [*https://doi.org/10.1016/j.livsci.2019.103899*](https://doi.org/10.1016/j.livsci.2019.103899).
- [*Animal*](https://www.sciencedirect.com/journal/animal), 2021, 14 up to 23 samples in local sheep from a semi-arid area, [*https://doi.org/10.1016/j.animal.2021.100193*](https://doi.org/10.1016/j.animal.2021.100193).
- *Genes*. 2022, 12 up to 74 from six Colombian sheep populations, [*https://doi.org/10.3390/genes13081415*](https://doi.org/10.3390/genes13081415).
- *Animal*, 2023, 48 individuals of Sumava and 37 individuals of Wallachian sheep (Czech Republic local sheep breeds), [*https://doi.org/10.1016/j.animal.2022.100690*](https://doi.org/10.1016/j.animal.2022.100690).

**======================= Reviewer 2**

The article entitled “Genome-wide study of linkage disequilibrium, population structure, and inbreeding in Iranian indigenous sheep breeds” focuses on the knowledge of linkage disequilibrium, genetic structure and genetic diversity in three Iranian local sheep breeds. This study is of wide interest to the community, but I have following concerns which needs to be addressed.

- The authors deeply appreciate the Reviewer for giving us the opportunity to revise and improve our manuscript. Thank you very much for your kind consideration and valuable comments.

**Comment 1:** In the introduction section, more breed information for Baluchi, Lori-Bakhtiari and Zel is needed.

- **Response 1:** The Introduction was altered to address the referee's comment.

**Comment 2:** why you state more information for QTL, GS, and LD in the introduction section? Please explain it. I personally feel that the current organizational structure of the introduction section is unreasonable, and more valuable information should be provided by the author.

- **Response 2:** Actually, we were interested to provide more explanation about the importance of linkage disequilibrium (LD) in genomic selection (GS) and QTL detection. However, additional explanations of GS has been removed and the introduction has been rewritten to clarify this point and to address the Referee's concern.

**Comment 3:** Line 92, please the name of database, or provide the accession number.

**Response 3:** The text was altered to the referee suggested rephrasing.

**Comment 4:** line 113, how many SNPs was remained? Please provide it in the text.

- **Response 4:** The remained SNPs was different per each breed and have been shown in details in Table 1.

**Comment 5:** line 118, provide the author name

- **Response 5:** The text was revised to address this point.

**Comment 6:** Line 200-202, please rewrite this sentence.

- **Response 6:** The sentence was rewritten to address the referee's concern.

**Comment 7:** Line 914, please fixed the subtitle.

- **Response 7:** The subtitle was fixed in Line 194.

**Comment 8:** Please write the results and discussion, respectively. The current write way is difficult for readers to clearly understand the author's intention.

**Response 8:** Thank you for the comment. The text of this section was revised substantially to present the results and discussion, respectively for each subtitles as the referee suggested.

**Comment 9:** Improve discussion and conclusion. More utility from this work should be signified.

**Response 9:** Your remark has been implemented to the text. The text of discussion was improved by using more references, and the conclusion was rewritten to address this invaluable comment.

6. PLOS authors have the option to publish the peer review history of their article ([what does this mean?](https://journals.plos.org/plosone/s/editorial-and-peer-review-process#loc-peer-review-history)). If published, this will include your full peer review and any attached files.

Reviewer #1: Yes: Arun Kumar De

Reviewer #2: No

=======================

The authors appreciate the Reviewers again for their invaluable comments. We think these comments helped us to improve the manuscript, significantly. We did our best and hopefully the changes have addressed the concerns.
